# Wound of the Heart

**Published:** 1900-12-15

**Authors:** 


### WOUND OF THE HEART.

In a recent number of the *Bulletin de la Soc. de Chirurg. de Paris* a case is recorded in which several wounds implicating the left ventricle of the heart, and also of the left lung, were treated by free exposure of the injured parts and closure of the wounds by sutures, with the result that complete cure took place. The patient was a soldier who had inflicted upon himself six punctured wounds between the third and seventh ribs in the precordial region. There were signs of pneumo-homothorax. A large flap including the anterior portions of the fourth, fifth, and sixth ribs was made and turned outwards by dividing the costal cartilages, and by section and fracture of the ribs involved at the outer portion of the flap. On this being done a large quantity of blood was found in the left pleural cavity, together with a superficial wound in the diaphragm, a wound of the anterior margin of the lung, and a wound about 1 centimetre in length in the pericardium. On enlarging this a penetrating wound about 12 centimetres in length was found on the anterior wall of the left ventricle. The wounds were closed with catgut, and the pericardial and pleural cavities were washed out with boiled water. The application of the suture to the ventricle was found difficult owing to the movement of the heart. Recovery was speedy and apparently complete.

